# Investigation of Chitosan-Based Hydrogels and Polycaprolactone-Based Electrospun Fibers as Wound Dressing Materials Based on Mechanical, Physical, and Chemical Characterization

**DOI:** 10.3390/gels11010039

**Published:** 2025-01-04

**Authors:** Barkin Aydin, Nihat Arol, Nimet Burak, Aybala Usta, Muhammet Ceylan

**Affiliations:** 1 Department of Mechanical Engineering, Engineering Faculty, Marmara University, 34854 Istanbul, Türkiye; aybarkin@hotmail.com (B.A.); nihat.arol@gmail.com (N.A.); nburak@borusan.com (N.B.); 2 Department of Mechatronics Engineering, Engineering Faculty, Istanbul Ticaret University, 34854 Istanbul, Türkiye; mceylan@ticaret.edu.tr

**Keywords:** PCL, electrospinning, boric acid, zinc oxide, hydrogel, chitosan

## Abstract

The aim of this project is to fabricate fiber mats and hydrogel materials that constitute the two main components of a wound dressing material. The contributions of boric acid (BA) and zinc oxide (ZnO) to the physical and mechanical properties of polycaprolactone (PCL) is investigated. These materials are chosen for their antimicrobial and antifungal effects. Additionally, since chitosan forms brittle hydrogels, it is reinforced with polyvinyl alcohol (PVA) to improve ductility and water uptake properties. For these purposes, PCL, BA, ZnO, PVA, and chitosan are used in different ratios to fabricate nanofiber mats and hydrogels. Mechanical, physical, and chemical characteristics are examined. The highest elastic modulus and tensile strength are obtained from samples with 6% BA and 10% ZnO concentrations. ZnO-decorated fibers exhibit a higher elastic modulus than those with BA, though BA-containing fibers exhibit greater elongation before breakage. All fibers exhibit hydrophobic properties, which help to prevent biofilm formation. In compression tests, CS12 demonstrates the highest strength. Increasing the PVA content enhances ductility, while a higher concentration of chitosan results in a denser structure. This outcome is confirmed by FTIR and swelling tests. These findings highlight the optimal combinations of nanofibrous mats and hydrogels, offering guidance for future wound dressing designs that balance mechanical strength, water absorption, and antimicrobial properties. By stacking these nanofibrous mats and hydrogels in different orders, it is expected to achieve a wound care material that is suitable for various applications. The authors encourage experimentation with different configurations of these nanofiber and hydrogel stackings to observe their mechanical behavior under real-life conditions in future studies.

## 1. Introduction

The skin serves as the body’s initial line of defense against external threats, protecting interior organs from any environmental damage [1]. It often experiences various types of wounds, including chronic and easily healed cuts, scrapes, scratches, and punctures. Wounds can also result from accidents or surgical procedures [2,3].

Wound healing is a dynamic biological process involving hemostasis, inflammation, proliferation, and remodeling [4]. Wound dressings aim for rapid healing with minimal scarring, preventing microbial infections. Materials must be absorbent, permeable, mechanically strong, moist, removable, replaceable, and affordable [5,6]. Modern dressings include hydrogels, hydrocolloids, alginates, foams, and nanofiber films [7,8]. Nanofiber mats and hydrogels are desirable materials in biomedical applications [9,10].

As was previously said, the qualities that make up an optimal wound dressing include its capacity to keep the site moist, guard against subsequent infections, encourage tissue regeneration, and improve the general effectiveness of wound healing. When the aforementioned elements are taken into account, hydrogels have a lot of potential as wound dressings. They are extensively employed in biomedical settings because of their high-water content, suppleness, and compatibility with biological systems [11,12]. In addition, they are utilized in tissue culture, drug release systems, and medical equipment, and they replenish or retain moisture in the surroundings in which they are utilized. They are perfect for wound dressings since their composition can be adjusted to match certain wound characteristics [13,14]. It should be highlighted, nonetheless, that hydrogels have a number of drawbacks including bacterial permeability, poor mechanical stability and relatively low mechanical strength, making them feeble and brittle [15,16].

Hydrogels are water-absorbent polymer networks that create a gel-like substance, allowing them to provide a supportive environment for cell growth and tissue regeneration. Their ability to retain moisture and mimic the properties of natural tissues makes them valuable in biomedical applications [17]. Hydrogels are developed using various natural or synthetic polymers. While natural polymers offer biomedical characteristics, synthetic polymers provide improved mechanical properties. Hydrogels that combine both natural and synthetic polymers tend to have more favorable characteristics, making them ideal for wound dressing applications [18].

Chitosan, one of the natural polymers to be used in wound dressing applications in the form of hydrogel, is a product of deacetylation of chitin. It is used in medicine for a variety of purposes, such as bandages, antibacterial agents, and medication transfer. Because of its advantageous characteristics, including biodegradability, biocompatibility, non-toxicity, antibacterial activity, biological adhesion, biological activity, hemostatic effects, high availability, and low-cost, chitosan is thought to be an extremely effective material for biomedical applications [19,20,21]. Additionally, being soluble in weak acids like acetic acid, it promotes faster wound healing through the activation of inflammatory cells, macrophages, and fibroblasts [22,23,24]. On the other hand, since hydrogels are also known for their low mechanical properties, they need to be strengthened by employing a range of crosslinking methods, such as enzymatic, chemical, and physical crosslinking. It is advised to chemically crosslink hydrogels using substances like formaldehyde or glutaraldehyde (GA) [25]. GA can be a good candidate for contributing to the resilience of the gel by introducing cross-linkages between chitosan chains. It is worth noting that due to the cytotoxic property of GA when used in a concentration above 0.1%, it should be exploited in the least amount possible in biomedical applications and other areas of use. Also, while mechanical properties are improved using crosslinker agents, they must ensure that the robust properties are not decreased. PVA is one of the synthetic polymers used to fabricate a composite hydrogel providing good mechanical strength [26,27]. PVA-based hydrogels have extensive applications in the food industry, biomedicine as drug carriers, tissue engineering scaffolds, wound dressings, artificial muscles and organs, and biosensors because of their excellent mechanical qualities, low toxicity, high water absorption, and strong biocompatibility [28].

Novel materials known as electrospun nanofiber membranes have a wide range of uses, one of which is as wound dressings. These membranes have a notable degree of microporosity, flexibility, and a high surface-to-volume ratio. Numerous benefits come with electrospun nanofiber wound dressings, which are made using electrospinning technology. First, they provide an ideal environment for cell adhesion, proliferation, migration, and differentiation because of their structural and biological similarities to the extracellular matrix (ECM). Second, the combination of natural polymers’ biocompatibility and synthetic polymers’ dependable mechanical strength is made possible by the polymer matrix employed in electrospinning [29]. Also, the mechanical properties of these fibers can be improved by introducing particulates into the structure.

Because of its mechanical qualities, high biocompatibility, high biodegradability (no harm to the environment), and ease of manufacturing, PCL is the hydrophobic polymer of choice for creating electrospun nanofibers [30]. In the meantime, PCL, a synthetic polymer, has a very slow rate of degradation and can control the rate at which drugs are released from scaffolds [31].

Zinc oxide (ZnO) microparticles exhibit significant antibacterial properties, primarily due to their ability to alter cell membrane permeability [32]. The mechanism involves the generation of reactive oxygen species that cause oxidative stress, damaging the bacterial cell membrane and leading to cell death [33]. These microparticles also demonstrate antifungal activity, inhibiting the growth of various Candida species. The primary mode of action against fungi is similar to bacteria, involving the production of ROS and disruption of cellular processes essential for fungal growth and survival [34].

Boric acid (BA) has long been recognized for its antibacterial and antifungal properties. It exhibits antibacterial activity by disrupting bacterial cell walls [35]. In terms of antifungal activity, BA is effective against various Candida species. The antifungal mechanism involves the inhibition of oxidative metabolism and disruption of cellular ergosterol production, essential for maintaining fungal cell membrane integrity. Additionally, BA interferes with the hyphal transformation of Candida species, a critical factor in fungal pathogenicity [34]. BA is more stable and significantly less toxic compared to metal nanoparticles. This renders BA a safer option for medical applications and environmentally friendly [36].

In the current study, hydrogels and nanofibers are prepared to make an effective combination for wound dressing application due to the aforementioned advantages of these materials. The study was conducted in two parts: fabrication of fibers and fabrication of hydrogels. Chitosan was therefore used in this project’s gel production process, and the brittle characteristic of chitosan was hypothesized to decrease when using PVA polymer. Also, ZnO and BA incorporated PCL fibers, which constitute the other component of wound dressing material, were fabricated using electrospinning. The mechanical characterization of these wound dressing materials, (Chitosan/PVA Hydrogels) and (PCL/ZnO/BA) was investigated in this work. In addition, other physical and chemical characterization tests were conducted, and optimum materials were selected for further wound dressing material design which is combination of both hydrogel and nanofiber.

## 2. Results and Discussion

### 2.1. Tensile Test Results of Electrospun Fibers

**Figure 1:**
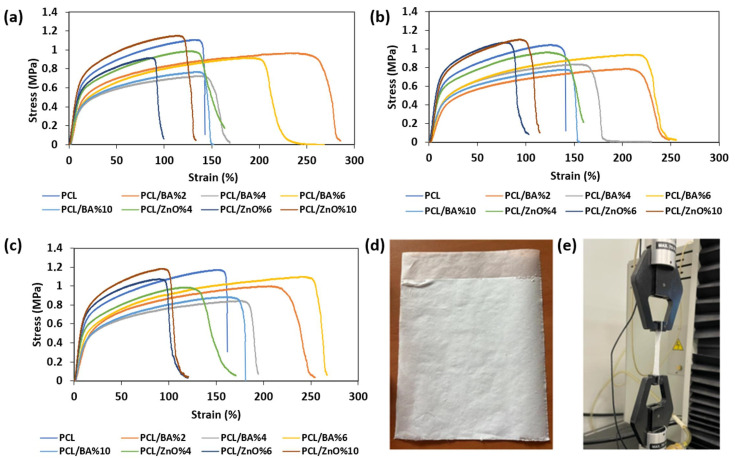
Stress–Strain graphs of specimens. (**a**) Stress–Strain graph of the first specimens. (**b**) Stress–Strain graph of the second specimens. (**c**) Stress–Strain graph of the third specimens. (**d**) Tensile test setup for fabricated fibers. (**e**) Electrospun mat with 6% ZnO.

**Figure 2:**
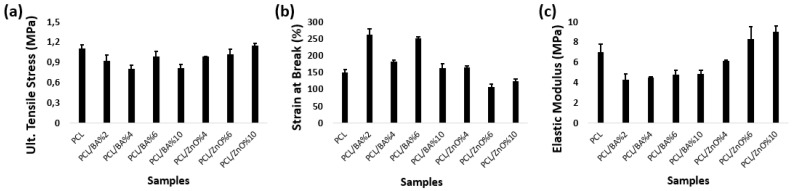
Tensile test results of electrospun nanofibers. (**a**) Ultimate Tensile stress (**b**) Strain values at failure (**c**) Elastic module values.

When incorporating BA particles into the fibers, there is an initial decrease in the elastic modulus compared to fibers containing only PCL. However, as the BA percentage in the fiber increases, the elastic modulus also increases (Figure 1). On the contrary, an increase in the amount of ZnO leads to an increase in the elastic modulus. This increase was statistically significant (*p <* 0.05). The highest values for ultimate tensile stress and elastic modulus were observed in fibers containing 10% ZnO. When looking at elongation percentages and the overall behavior of the fibers, all of them exhibit ductile properties. Nonetheless, fibers containing BA exhibit greater elongation compared to those containing ZnO, and these fibers with 6% BA showed the highest tensile strength following the fibers with 10% ZnO, and greatest elongation (Figure 2). ANOVA results confirmed that changes in tensile strength and strain were statistically significant (*p <* 0.05). There also was a statistically significant difference found between the BA and ZnO decorated groups (*p* < 0.05). The observed increase in both elastic modulus and ultimate tensile strength in fibers containing 10% ZnO is attributed to the role of ZnO as a reinforcing agent within the microstructure of the PCL nanofibers. This enhancement in mechanical properties is likely due to the dispersion of ZnO within the polymer matrix. FTIR analysis showed no new chemical bond formation between PCL and ZnO, suggesting that the reinforcement is primarily physical [37]. It was discovered that the inclusion of particles can considerably enhance the elastic modulus and yield fiber strength. Tjong et al. [38] related the mechanism of strength enhancement to the effect of nanoparticles on the restricted movement of polymer chains. According to Bugnicourt et al. [39], the interaction between polymer molecules and nanoparticles during deformation greatly reduces their mobility, hence promoting an enhancement in elastic modulus.

These findings suggest that 6% BA and 10% ZnO can be better options in the enhancement of mechanical properties of nanofibers, meanwhile providing antibacterial and antibacterial effects. Also, these fibers can be implemented into the design and mechanical characterization study of the future sandwich structured fiber–hydrogel wound dressing materials study.

### 2.2. Morphological Characterization of Nanofiber Mats

**Figure 3:**
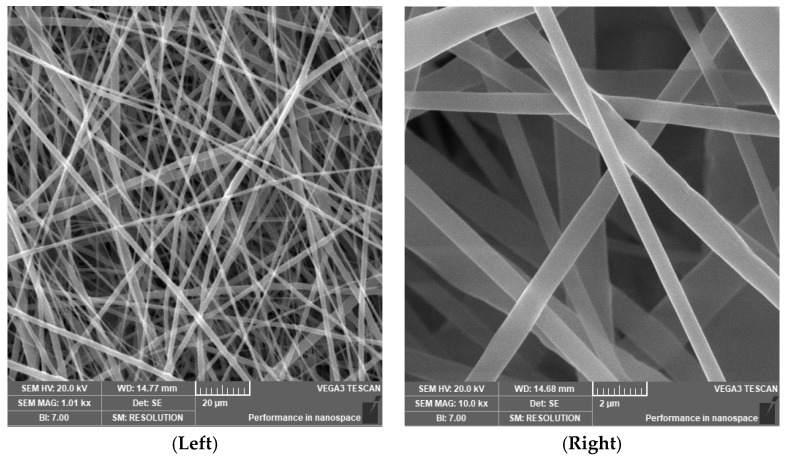
SEM image of surface morphology. 1 kx magnification (**Left**), 10 kx magnification (**Right**).

SEM analysis of electrospun PCL nanofibers exhibited a nanofibrous surface texture. The images are presented in Figure 3.

### 2.3. Contact Angle Test Results

**Figure 4:**
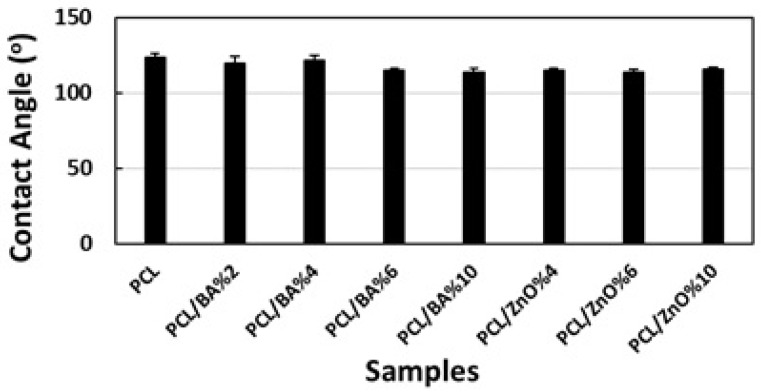
Contact angle values of electrospun nanofiber with different amounts of BA and ZnO.

**Figure 5:**
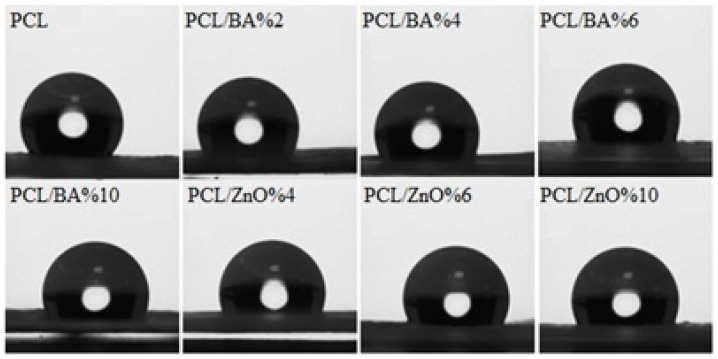
Contact angle images of electrospun nanofibers at the first contact.

Contact angle study indicated that all the fibers exhibit hydrophobic characteristics. The addition of microparticles has led to a reduction in the contact angle, but they still maintain their hydrophobic nature, as the contact angle values always remain above 90° (Figure 4). Both ZnO and BA have hydrophilic characteristics, while PCL displays hydrophobic properties. The reason all fibers exhibit hydrophobicity is due to the large proportion of PCL in the composition. As the percentage of particles increases, hydrophobicity decreases, which can be attributed to this factor. Contact angle images of drops on fiber surfaces can also be seen in Figure 5. The importance of hydrophobicity in electrospun mats for wound dressing applications comes from the fact that hydrophobic dressings limit the formation and growth of biofilm in the wound by a physical action, and as a result the risk of antibiotic resistance is greatly minimized [40]. In this sense, desired hydrophobicity for the mat material part of the dressing material was achieved for all formulations. The ANOVA study showed that there was no significant difference found between the BA and ZnO decorated fiber groups since *p* value was greater than 0.05). However, the addition of BA and ZnO microparticles in various percentages had a statistically significant effect on contact angle values of PCL fibers.

**Figure 6:**
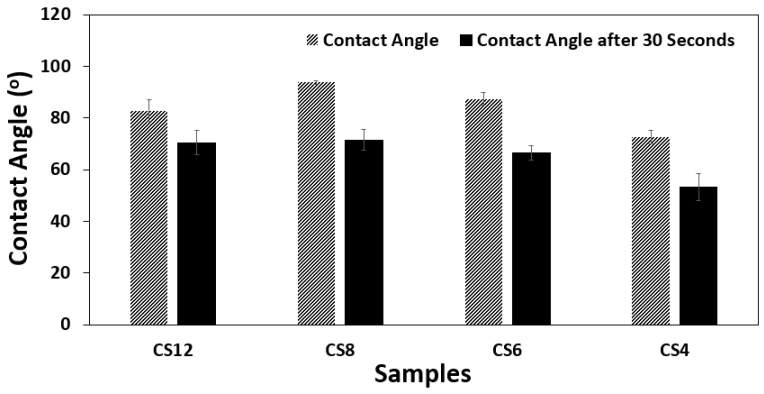
Contact angle values of hydrogels. Initial contact angle (dashed bars), contact angle of the same drops after 30 s (solid bars).

**Figure 7:**
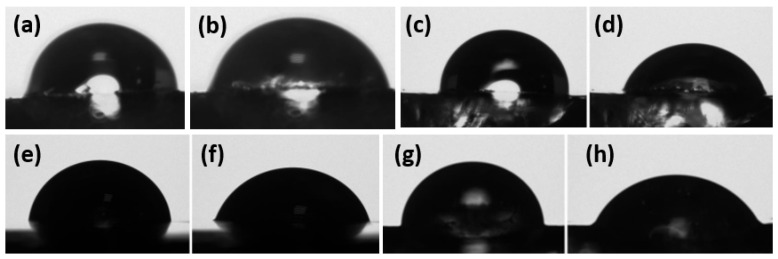
Contact angle images of hydrogels. (**a**) CS12, (**b**) CS12 after 30 s, (**c**) CS8, (**d**) CS8 after 30 s, (**e**) CS6, (**f**) CS6 after 30 s, (**g**) CS4, (**h**) CS4 after 30 s.

In wound dressing applications, hydrophilic behavior is desired from hydrogel material to keep the environment moist, and absorb the excess fluid generated in the wound area. For this purpose, contact angle studies were also conducted for hydrogel samples prepared with various chitosan and PVA amounts. The contact angles were measured for hydrogel samples both initially, when the drop touched the gel, and after 30 s. Fabricated hydrogels exhibited hydrophilic nature, and water uptake behavior can be observed from the drop in contact angle values after 30 s, which indicates the high water absorption property of the gels (Figure 6 and Figure 7). Due to the abundance of hydroxyl groups in the PVA molecule, with increasing the PVA ratio, the hydrophilicity has improved. This fact can be observed from contact angle results in addition to water uptake properties of the hydrogels. Also, FTIR and water uptake properties of the hydrogels suggest the same results.

### 2.4. Compression Test Results of Hydrogels

**Figure 8:**
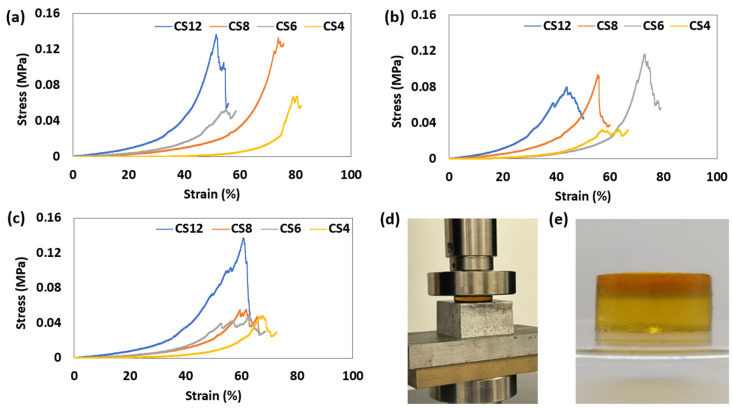
Stress–Strain graphs of specimens for compression test. (**a**) Stress–Strain graph of the first specimen. (**b**) Stress–Strain graph of the second specimen. (**c**) Stress–Strain graph of the third specimen. (**d**) Compression test setup for fabricated hydrogels. (**e**) A sample for compression test.

**Figure 9:**
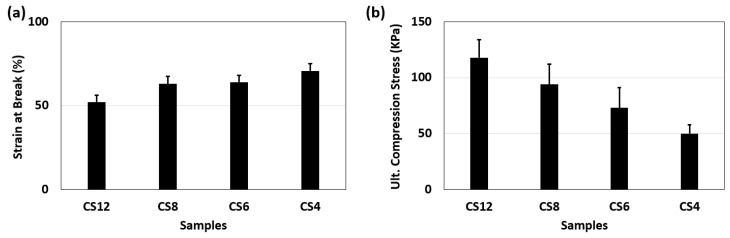
Compression test results of hydrogels. (**a**) Strain values at failure. (**b**) Ultimate compression stress.

The compression test results clearly suggest that the CS12 hydrogel exhibits the highest strength among the listed hydrogels. It was noticed that a decrease in the chitosan content, coupled with an increase in PVA, leads to a decrease in the ultimate compressive stress. However, the ductility of gels increases as the amount of PVA rises. In summary, as the quantity of chitosan rises, the material becomes sturdier and more prone to brittleness. Conversely, an increase in PVA content results in reduced strength with reduced brittleness, thus meaning that increased elongation at breakage (Figure 8 and Figure 9). These findings suggest that PVA might inhibit the cross-linking of chitosan, a deduction supported by the FTIR test results. Additionally, the higher hydrophilicity of PVA compared to chitosan likely increases the samples’ fluidity, leading to a less robust hydrogel structure. However, these differences in the compression test were not statistically significant since the *p*-value was found to be greater than 0.05. This suggests that more datasets should be collected for future experimentation.

### 2.5. FTIR Analysis

**Figure 10:**
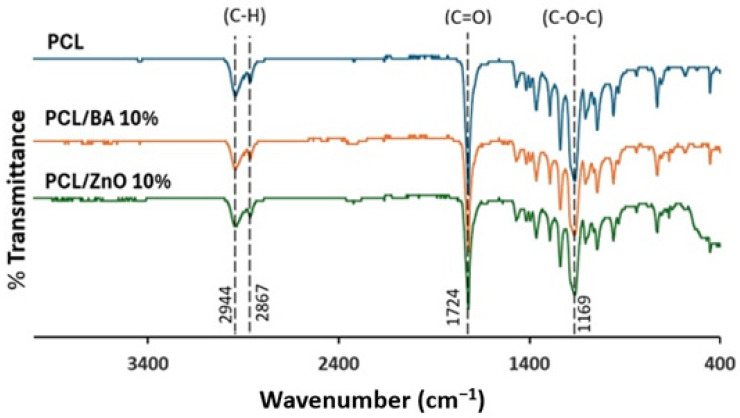
FTIR Spectra of electrospun nanofiber samples PCL, PCL/BA10%, and PCL/ZnO10%.

The chemical interactions of PCL, BA, and ZnO were investigated using FTIR spectroscopy. This analysis proved that the addition of BA and ZnO into PCL solution did not cause any structural change in PCL, suggesting that there was no chemical interaction between these chemicals (Figure 10). Distinct peaks at 1724 and 1169 cm^−1^ are assigned to –C=O and C–O–C bonds (stretching) in ester bonds for PCL, respectively. Furthermore, peaks at 2944 and 2867 cm^−1^ are assigned to anti-symmetric methylene–oxygen (CH2–O) and symmetric methylene groups (CH2–), respectively [41], indicating the presence of common functional groups in the polymer. Except for the PCL peaks, no new bonds were observed in PCL/BA or PCL/ZnO, indicating that no chemical reaction occurred during the mixing of these materials. Furthermore, there was no shift in peaks in PCL/BA or PCL/ZnO with respect to PCL, indicating that the relative decreases in transmittance were caused by a progressive decline in PCL concentration due to modest amounts of added BA and ZnO. Considering the desired antifungal and antibacterial activity of BA and ZnO for ROS formation and cell membrane disturbance mechanisms, maintaining these chemical characteristics makes these electrospun fiber mats a potential material in wound dressing applications.

**Figure 11:**
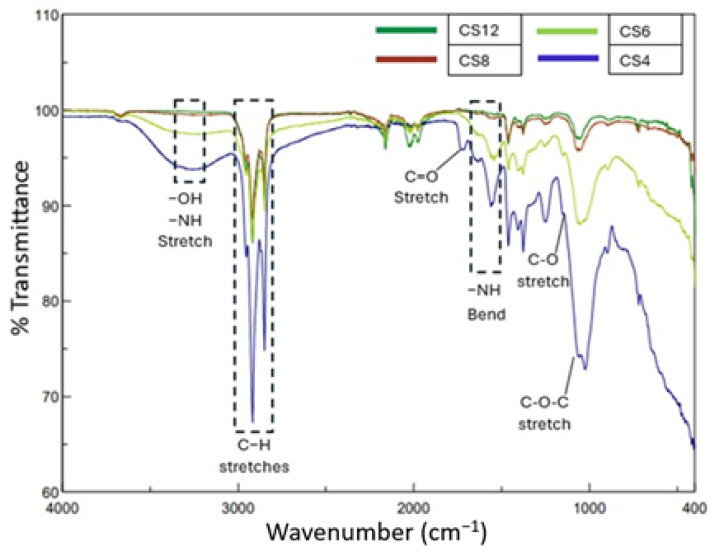
FTIR Spectra of samples CS12, CS8, CS6, and CS4.

Figure 11 shows the FTIR spectra of hydrogels made of different proportions of chitosan and PVA solutions. All significant peaks associated with the hydroxyl and acetate groups were observed. The broad bands between 3500 and 3100 cm^−1^ are assigned to stretching −OH (inter/intra-molecular hydrogen bonds of hydroxyl groups) and N−H stretching. It is evident that a higher PVA/GA ratio led to less crosslinking and, as a result, an increase in the amount of accessible hydroxyl groups. The vibrational band observed between 2830 and 3000 cm^−1^ refers to the stretching C–H from alkyl groups.

The peak around 1557 cm^−1^ was the vibration peak of –NH2 on chitosan, suggesting that the reaction completely occupied the –NH_2_ bending groups on the samples CS12 and CS8 [42]. Absences of two absorption spectra in chitosan hydrogel around 1655 cm^−1^ and 1600 cm^−1^, which are related to stretching vibration of –NHCO– (type I amide) and –NH_2_ bending group (type II amide), respectively, is an indicator of the cross-linkage between chitosan and GA. However, the rise in the intensities of these two peaks with the addition of PVA solution led to an increase. This suggests that the PVA addition hindered the chemical interaction between chitosan and GA. Moreover, a new peak at 1734 cm^−1^ is associated with –C=O stretching vibration of the carbonyl groups in aldehyde for CS4 which has the highest proportion of PVA solution. Detection of this peak also indicated that there might be unreacted GA in the mixture which did not react with amine groups of chitosan and hydroxyl groups of PVA, and form acetal bonds.

### 2.6. Swelling Test

**Figure 12:**
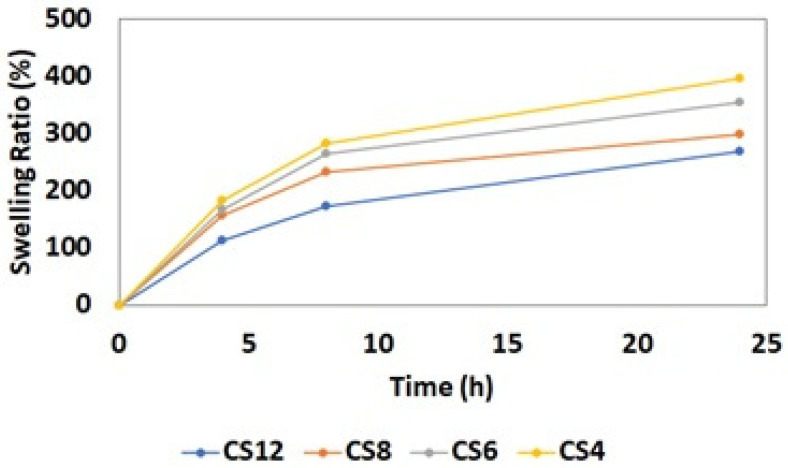
Evaluation of swelling degree of the CS/PVA hydrogels crosslinked with GA.

The swelling test conducted on hydrogels revealed that as the quantity of chitosan in the hydrogel decreases, the swelling ratio increases gradually (Figure 12). This occurrence is attributed to the crosslinking agent GA, bonding with larger amounts of chitosan as chitosan concentration increases. This bonding creates a denser structure, reducing the available space within the hydrogel for water absorption. It can also be attributed to the hydrophilic nature of PVA. Since PVA chains have plenty of hydrophilic hydroxyl groups (–OH), increasing the PVA content in the mixture resulted in a more hydrophilic structure and enhanced the water uptake properties. The results from the contact angle test also support this correlation observed in the swelling test (Table 1).

## 3. Conclusions and Future Remarks

The greatest elastic modulus and tensile strength among all the formulated electrospun fibers were achieved from the samples with 6% BA and 10% ZnO concentration. It was observed that the elastic modulus increased when the amount of ZnO and BA increased, and ZnO decorated fibers demonstrated a higher modulus of elasticity compared to BA decorated fibers. When the elongation percentages and graphic shapes were examined, it was observed that all fibers showed ductile properties. However, the fibers containing BA elongated more than those containing ZnO until breakage. Along with the information gained from the FTIR analysis, it is observed that PCL did not form new bonds with either BA or ZnO. Therefore, the difference in elastic modulus and ultimate tensile strength between BA and ZnO containing nanofibrous mats might be attributed to the dispersion quality of the microparticle within the polymer matrix. It was clearly seen that all fibers showed hydrophobic properties. Microparticle addition has decreased the contact angle slightly; however, they maintained the hydrophobic behavior since the majority of the compound is PCL. This showed that possible biofilm formation can be hindered by reducing adhesion and antimicrobial resistance. The results of the compression tests of hydrogels showed that CS12 hydrogel has the highest strength. It was observed that when the amount of chitosan decreased and the amount of PVA increased, the ultimate compressive stress also decreased. However, the ductility of the gel increased when PVA increased. To summarize, as the amount of chitosan increases, the material becomes more robust and brittle, and as the amount of PVA increases, the material becomes weaker and less brittle. Based on this result, it can be concluded that PVA might have blocked the cross-linking of chitosan. This inference was also proved by the FTIR test results observing the increase in the intensity of unbonded hydroxyl and amine groups. As a result of the swelling test for hydrogels, it was seen that by gradually decreasing the amount of chitosan in the hydrogel, the swelling ratio increases. The reason for these phenomena might be due to the crosslinking agent GA bonding with increasing amounts of chitosan, forming a denser and more intense structure thus leaving less space inside the hydrogel for water absorption. Contact angle and swelling test results also approve this phenomenon for the result of the swelling test. Based on these outcomes of the performed tests, future studies regarding the stacking sequence of electrospun nanofiber mat and hydrogel will be conducted in a way to provide best mechanical strength, water absorption, and antimicrobial effect properties.

This study enables us to evaluate which electrospun fibers and hydrogels possess the most suitable properties for sandwich-like wound dressings applications. Furthermore, it allows us to determine the optimal configuration for layering these nanofibers and hydrogels to maximize their functional effectiveness.

## 4. Material and Methods

### 4.1. Materials

Chloroform, glacial acetic acid, and acetonitrile were purchased from ISOLAB (Türkiye). PCL with Mw: 80,000 was obtained from Sigma-Aldrich (Türkiye). Technical grade ZnO and BA with the densities of 5.61 g/cm^3^ and 1.44 g/cm^3^ were purchased from Balmumcu Kimya (Türkiye). 95% deacetylated Chitosan was purchased from Biyopol (Türkiye). 50% purity GA was purchased from Alfasol (Türkiye). 99% hydrolyzed PVA with M_w_ 89,000–98,000 was kindly gifted from Istanbul Ticaret University. All samples were prepared using deionized water.

### 4.2. Methods

#### 4.2.1. Preparation of Samples for Electrospinning

Initially, 15% (*w*/*w*) PCL solution was prepared by dissolving PCL in chloroform and acetonitrile solution at the ratio of 4:1, respectively. Later, other samples also were prepared adding different proportions of BA (2%, 4%, 6%, and 10% of PCL amount), with the remainder being PCL. A similar procedure was followed for ZnO solutions. The proportions were selected using a systematic incremental approach. To clearly observe the effect of the added particles, the increments were selected in 2% intervals. The resulting mixtures were stirred at 200 rpm for 24 h using a magnetic stirrer at a temperature of 50 °C. Subsequently, they were left at room temperature for 2–3 h to allow air bubbles to dissipate.

#### 4.2.2. Preparation of Hydrogels

To produce chitosan solution, 4 g of chitosan were dissolved in 100 milliliters of 1% aqueous acetic acid solution, while continuously stirring at a temperature of 75 °C. Subsequently, 2% PVA solution was prepared in deionized water at 90 °C until complete dissolution. Then, these mixtures were left to rest for 2 h to eliminate air bubbles. After resting, they were cooled in the refrigerator. The cold solutions of chitosan (CS) and PVA were blended at specific proportions, after which a 2 mL of 2% GA solution was introduced drop wisely to facilitate crosslinking. The resulting mixture was poured into molds and left at room temperature for a duration of 24 h to complete the reaction process. The hydrogels were formulated using a total volume of 12 mL of a combined PVA–CS solution. In this formulation, a method was employed where the concentration of chitosan was progressively increased by 2 mL until 8 mL and then increased by 4 mL to obtain a 0 mL PVA containing gel. The exact ratios used are detailed in Table 2.

#### 4.2.3. Electrospinning Process

The electrospinning procedure was conducted using the Inovenso NE 200 Nanospinner (USA), applying a voltage of 25 kV, a flow rate of 1 mL/h, and a cylinder collector rotating at 500 rpm. The distance between the nozzle and the collector was set at 25 cm.

#### 4.2.4. Material Characterization

The Instron 4411 Tensile Strength Tester machine was utilized for conducting the tensile tests. For the tensile testing process, samples measuring 60 × 10 mm were obtained from the electrospun mats. The samples were stretched at a rate of 30 mm/min until they broke, with a gauge length of 40 mm. Tests were repeated 3 times for each sample.

The compression testing was conducted using the “Shimadzu AGS-X 50 kN” machine (Japan), with a chosen strain rate of 5 mm/min. Cylindrical-shaped hydrogel specimens measuring 12 mm in height and 30 mm in diameter were used. Each hydrogel underwent three separate tests under room temperature conditions.

Fourier Transform Infrared (FTIR) spectroscopy analysis was performed to characterize the presence of certain functional groups in the samples. Chitosan, PVA, and other electrospun fiber samples were analyzed with the JASCO-4X Fourier Transform Infrared Spectrometer (Japan). Attenuated total reflectance (ATR) spectroscopy, which is one of the FTIR techniques, was used to characterize the structure of the prepared samples. FTIR spectra were collected with wavenumber ranging from 4000 to 400 cm^−1^ with the resolution of 4 cm^−1^. Major vibration bands were found and linked to the primary chemical groups after the FTIR spectra were normalized.

To assess the hydrophobicity and wettability of the nanofiber mats and hydrogels, water contact angles were determined. The contact angle measurements were conducted using the KSV CAM 101 (Finland) goniometer, the average water droplet volume was 10 μL, and all samples were tested 3 times.

For the initial examination of biodegradable materials, fluid absorption experiments are crucial. To assess fluid uptake, previously dried gels were placed in a 1% (*w*/*v*) saline solution and incubated at room temperature for a period of 24 h. During this incubation, the weights of the gels were checked at the 4, 8, and 24 h marks. The use of saline solution aimed to replicate conditions like sweat. Following varying durations of immersion, the specimens were delicately extracted from the medium, and following the removal of surplus water using filter paper, their weight was measured to ascertain the wet weight in relation to the immersion duration. Swelling ratio (SR) is given by Equation (1):

(1) $$SR\;\left(\%\right)=\frac{W_{S}-W_{D}}{W_{D}}*100$$

The surface morphology of PCL electrospun fibers was examined with a scanning electron microscope (SEM) (Tescan Vega3, Czechia). Before scanning, the fiber mat was Au-Pd sputtered to ensure conductivity.

#### 4.2.5. Statistical Analysis

A sample size of 3 has been used in this study. The data were analyzed using one-way and two-way analysis of variance (ANOVA). The value of *p* < 0.05 was considered statistically significant.

## Figures and Tables

**Table 1:** Results of swelling test.

Hours (h)	Swelling Ratios (%)			
	CS12	CS8	CS6	CS4
4	114	157.1	167.9	186.5
8	172.8	234.2	264.9	280.8
24	268.5	300	355.2	395.2

**Table 2:** Content ratios of hydrogels.

Sample	Solution Amounts (mL)		
	%4 CS	%2 PVA	%2 GA
CS12	12	0	2
CS8	8	4	2
CS6	6	6	2
CS4	4	8	2

## Data Availability

Additional datasets generated during the current study are available from the corresponding author on reasonable request.
